# The anti-SARS-CoV-2 monoclonal antibody, bamlanivimab, minimally impacts the endogenous immune response to COVID-19 vaccination

**DOI:** 10.1126/scitranslmed.abn3041

**Published:** 2022-06-09

**Authors:** Robert J. Benschop, Jay L. Tuttle, Lin Zhang, Josh Poorbaugh, Nicole L. Kallewaard, Peter Vaillancourt, Melissa Crisp, Thi Ngoc Vy Trinh, Joshua J. Freitas, Stephanie Beasley, Montanea Daniels, Natalie Haustrup, Richard E. Higgs, Ajay Nirula, Myron S. Cohen, Mary Marovich

**Affiliations:** ^1^ Eli Lilly and Company, Indianapolis, IN 46225, USA.; ^2^ Institute of Global Health and Infectious Diseases, University of North Carolina at Chapel Hill, NC27599, USA.; ^3^ Division of AIDS, National Institute of Allergy and Infectious Diseases, National Institutes of Health, Rockville, Maryland 20850, USA

## Abstract

As the coronavirus disease 2019 (COVID-19) pandemic evolves and vaccine rollout progresses, the availability and demand for monoclonal antibodies for the prevention and treatment of severe acute respiratory syndrome coronavirus 2 (SARS-CoV-2) infection are also accelerating. This longitudinal serological study evaluated the magnitude and potency of the endogenous antibody response to COVID-19 vaccination in participants who first received a COVID-19 monoclonal antibody in a prevention study. Over the course of six months, serum samples were collected from a population of nursing home residents and staff enrolled in a clinical trial who were randomized to either bamlanivimab treatment or placebo. In an unplanned component of this trial, a subset of these participants was subsequently fully vaccinated with two doses of either SpikeVax (Moderna) or Comirnaty (BioNTech/Pfizer) COVID-19 mRNA vaccines. This post-hoc analysis assessed the immune response to vaccination for 135 participants without prior SARS-CoV-2 infection. Antibody titers and potency were assessed using three assays against SARS-CoV-2 proteins that bamlanivimab does not efficiently bind to, thereby reflecting the endogenous antibody response. All bamlanivimab and placebo recipients mounted a robust immune response to full COVID-19 vaccination, irrespective of age, risk-category, and vaccine type with any observed differences of uncertain clinical importance. These findings are pertinent for informing public health policy with results that suggest that the benefit of receiving COVID-19 vaccination at the earliest opportunity outweighs the minimal effect on the endogenous immune response due to prior prophylactic COVID-19 monoclonal antibody infusion.

## Teaser

Individuals infused with an anti-SARS-CoV-2 antibody demonstrated a robust immune response to subsequent COVID-19 vaccination.

## INTRODUCTION

In response to the coronavirus disease 2019 (COVID-19) pandemic, many prophylactic and therapeutic treatments were rapidly developed to target the highly pathogenic severe acute respiratory syndrome coronavirus 2 (SARS-CoV-2) (*1*, *2*). Antibodies that target the receptor binding domain (RBD) of the spike protein of SARS-CoV-2 are essential for protection against COVID-19 (*3*, *4*); these antibodies reduce SARS-CoV-2 viral load, which is correlated with disease severity (*5*–*7*). Active immunity against COVID-19 develops when endogenous RBD-neutralizing antibodies are elicited following exposure to a pathogenic agent, such as SARS-CoV-2 or a COVID-19 vaccine (*3*, *4*); passive immunity is conferred through the administration of exogenous antibodies, such as monoclonal antibodies (mAbs), that precisely target and bind to the RBD. Several clinically active COVID-19 mAbs provide immediate protective immunity that persists for as long as the antibody concentration exceeds that required for neutralization of the virus (*8*–*12*). Bamlanivimab was the first COVID-19 mAb to be granted Emergency Use Authorization (EUA) in November 2020 by the U.S. Food and Drug Administration (FDA), but was later revoked in April 2021 due to the increase of SARS-CoV-2 viral variants that were resistant to bamlanivimab alone (*11*, *13*). Vaccine-induced protection develops over time, often requiring multiple doses of vaccine, but offers clear advantages by eliciting a broader polyclonal immune response and establishing immunological memory for durable immunity (*14*, *15*).

There are currently insufficient safety and efficacy data with COVID-19 vaccines in individuals who have previously received COVID-19 mAbs; in the absence of data, both the World Health Organization (WHO) and the Centers for Disease Control and Prevention (CDC) recommend the deferral of vaccination for 90 days following mAb treatment and more recently, if mAbs were received for post-exposure prophylaxis (PEP), the CDC now recommends vaccine deferral for 30 days (*16*, *17*). In order to avoid unnecessary delays for individuals seeking vaccination and to inform public health policy, it is critical that we understand any effect that therapeutic mAbs have on the subsequent vaccine-induced immune response.

Vaccine efficacy against COVID-19 is correlated with the elicitation of antibodies, and accordingly serological assays are critical tools for monitoring the longitudinal endogenous antibody responses following COVID-19 treatments or SARS-CoV-2 infection (*18*–*20*). In a prospective treatment case study, an individual who was treated with an anti-SARS-CoV-2 mAb for symptomatic SARS-CoV-2 infection and received mRNA COVID-19 vaccination more than 40 days thereafter, exhibited comparable post-vaccine antibody responses to SARS-CoV-2 RBD for SARS-CoV-2 variants (including Alpha, Beta, and Gamma), to other participants who had not received an anti-SARS-CoV-2 mAb and were vaccinated following confirmed SARS-CoV-2 infection (*21*). However, larger studies are required to assess the duration of exogenous anti-SARS-CoV-2 mAbs in individuals with COVID-19 and whether these mAbs interfere with a subsequent immune response to a later COVID-19 vaccine. Additional studies are also required to assess the potential impact of prophylactic treatment with anti-SARS-CoV-2 mAbs on the specificity, magnitude, functionality, and duration of the endogenous antibody response to SARS-CoV-2 infection or COVID-19 vaccination. Here we study the question of whether the presence of prophylactic mAbs in SARS-CoV-2-naïve individuals interferes with endogenous immune responses to vaccination. During the BLAZE-2 clinical trial, participants received either bamlanivimab mAb or placebo and, in an unplanned component, received COVID-19 vaccine doses at different timepoints as determined by the U.S. vaccination program. Here, we present the results from a post-hoc analysis of immune responses to full COVID-19 vaccination with either SpikeVax (mRNA-1273, Moderna) or Comirnaty (BNT162b2, BioNTech/Pfizer) mRNA vaccines following passive immunization with bamlanivimab mAb administered as a COVID-19 prevention intervention for participants who were residents or staff of U.S. skilled nursing and assisted living facilities (*22*).

## RESULTS

### Participant Characteristics

The BLAZE-2 (NCT04497987) clinical trial was a phase 3, randomized, double-blind, placebo-controlled, single-dose study to evaluate whether bamlanivimab prevented SARS-CoV-2 infection in staff and residents of skilled nursing and assisted living facilities with a high risk of SARS-CoV-2 exposure. This post-hoc analysis included a total of 498 samples from 135 SARS-CoV-2-naïve participants who received either bamlanivimab (4200 mg) or placebo (day 1) during the BLAZE-2 prophylaxis study and were subsequently fully vaccinated within the scheduled serum sampling period of the trial (day 169). The CDC describes an individual as fully vaccinated 2 weeks after the second COVID-19 vaccine dose in a two-dose series, such as for Comirnaty or SpikeVax (*23*).

Participants received the first COVID-19 vaccine dose at different timepoints (ranging from 43 to 127 days, median 67 days) following bamlanivimab or placebo infusion. A total of 95 participants (70%) received the first vaccine dose within 90 days of the bamlanivimab or placebo infusion. Most participants received the second COVID-19 vaccine dose following the recommended interval specified in the EUA factsheet for each vaccine (21 and 28 days later for Comirnaty and SpikeVax, respectively) (*24*, *25*). A total of 96 participants (71%) received the Comirnaty vaccine and 39 participants (29%) received the SpikeVax vaccine. The baseline characteristics of the participants included in this analysis are shown in Table 1.

**Table 1.** Baseline characteristics of participants (n=135) included in post-hoc analysis.

Characteristics	Bamlanivimab (4200 mg)		Placebo	
	Residents N=22	Staff N=51	Residents N=14	Staff N=48
Age, Median (range)	63 (31 - 95)	43 (20 - 74)	82 (63 - 93)	44 (19 - 67)
Sex, No. (%) Male Female	10 (45%) 12 (55%)	11 (22%) 40 (78%)	5(36%) 9 (64%)	4 (8%) 44 (92%)
High-risk of severe COVID-19, No. (%)	22 (100%)	24 (47%)	14 (100%)	21 (44%)
Vaccine, No. (%) SpikeVax Comirnaty	8 (36%) 14 (64%)	10 (20%) 41 (80%)	7 (50%) 7 (50%)	14 (29%) 34 (71%)

High-risk categorization criteria (*13*)

The median age of staff participants (n=99) was 43 years compared with resident participants (n=36) who had a median age of 72 years. Of the 99 staff participants, 6% were 65 years or older. A total of 81 participants (60%) met the criteria for high-risk of developing severe COVID-19, which included 100% of the residents and 45% of the staff. The criteria for classifying high-risk of severe COVID-19 for this post-hoc analysis have been described previously (*13*).

### Full COVID-19 vaccination elicited SARS-CoV-2-binding endogenous antibody responses following anti-SARS-CoV-2 mAb infusion.

**Fig. 1.**
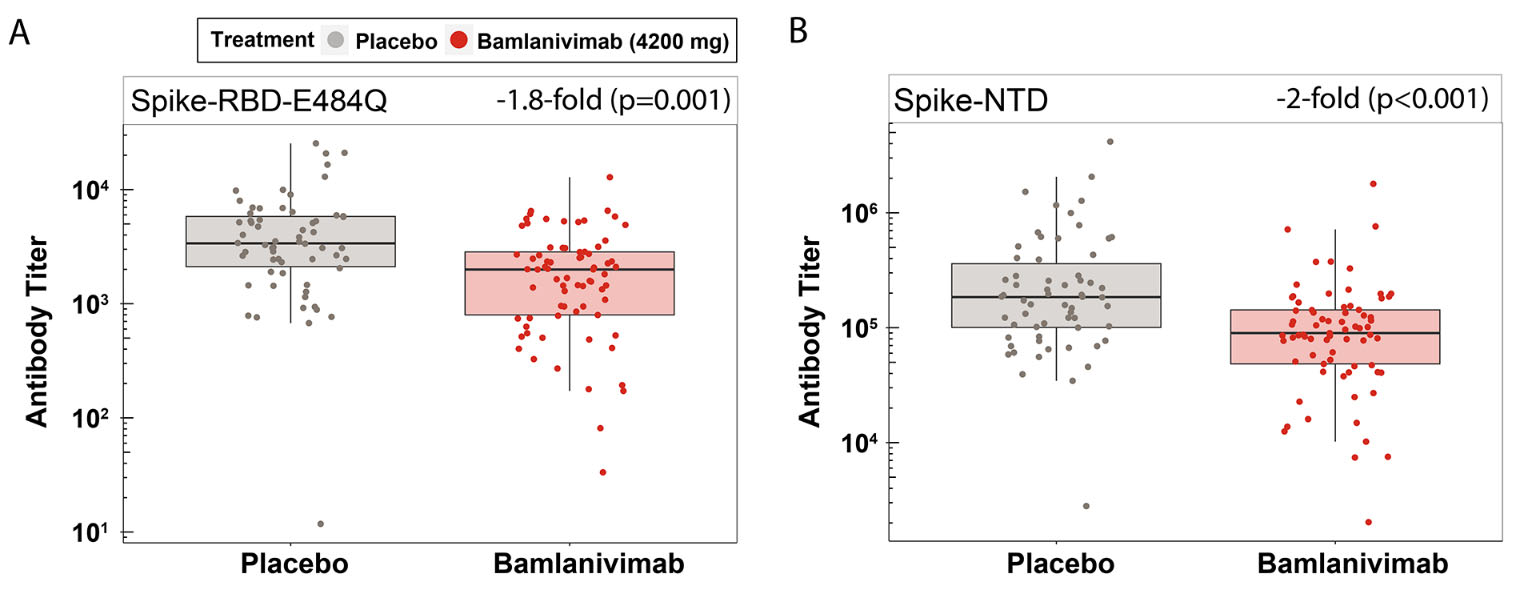
Bamlanivimab minimally impacts on endogenous antibody response to full COVID-19 vaccination. Binding antibody titers against (**A**) spike-RBD-E484Q and (**B**) spike-NTD are shown for participants who had received either placebo (n=62) or bamlanivimab (n=73) infusion and were subsequently fully vaccinated (SpikeVax or Comirnaty) against COVID-19. Antibody titers were rescaled after adjusting for covariates. Boxes and horizontal bars denote the interquartile range (IQR) and the median, respectively. Length of whiskers corresponds to 1.5 times the IQR. Statistical analysis was done using a linear model (two-sided test with α level of 0.05).

A multiplex custom assay was performed on serum samples obtained from fully vaccinated participants (n=135) to measure the magnitude of the binding antibody response. The assay was performed against the spike-RBD carrying the E484Q alteration (spike-RBD-E484Q) and the spike N-terminal domain (spike-NTD) (table S1). Since the epitope for bamlanivimab lies within the spike RBD (*26*, *27*) and bamlanivimab does not efficiently bind to the RBD with alterations at residue E484 (*28*, *29*) or to NTD, antibody titers against these two SARS-CoV-2 proteins reflect the endogenous antibody response. Compared with placebo, treatment with bamlanivimab resulted in a 1.8-fold (p=0.001) and 2.0-fold (p<0.001) lower antibody titer against spike-RBD-E484Q (Fig. 1A) and spike-NTD (Fig. 1B), respectively, using a least square means comparison.

**Fig. 2.**
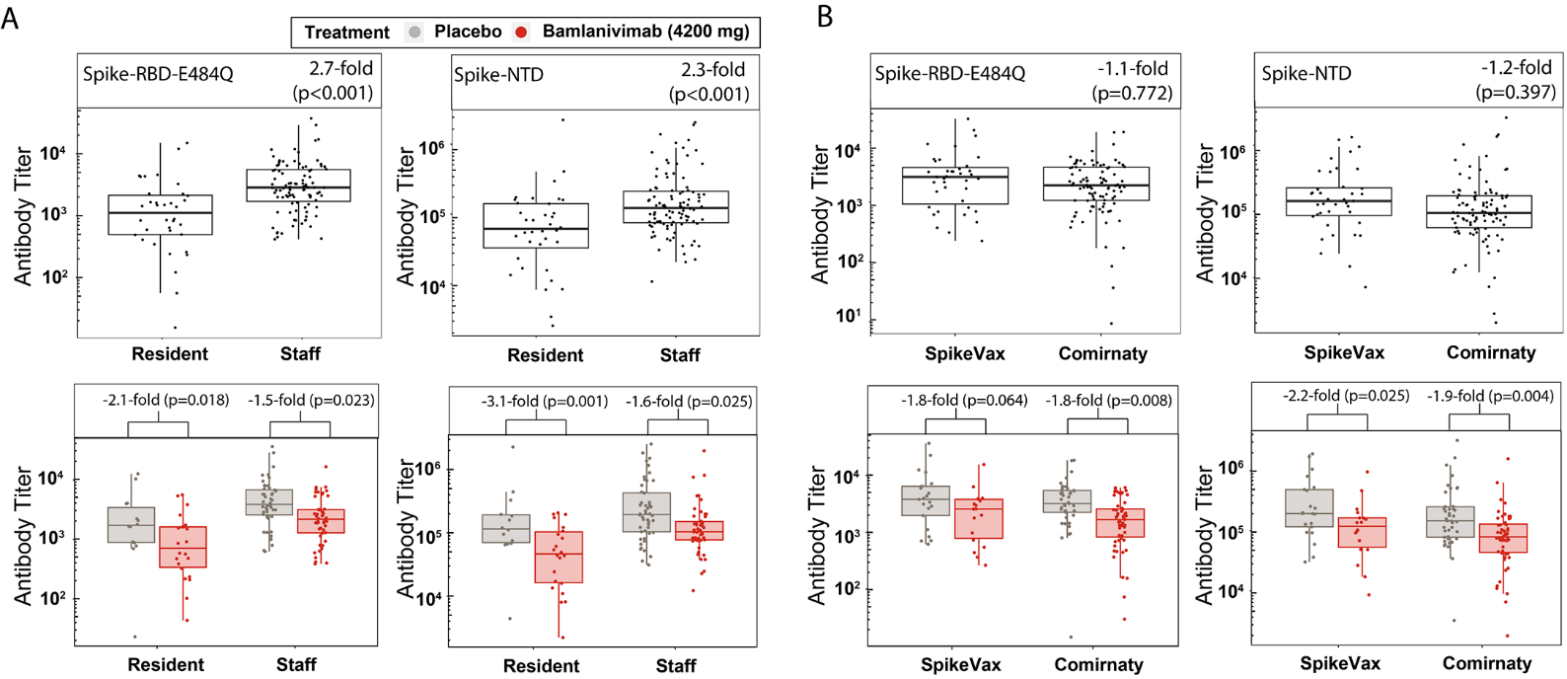
Prior bamlanivimab infusion did not disproportionately affect endogenous antibody response to full COVID-19 vaccination in residents and staff or SpikeVax- and Comirnaty-treated patients. (**A**) Binding antibody titers against spike-RBD-E484Q and spike-NTD are shown for samples from fully vaccinated participants who were staff (n=99) or resident (n=36) (top) and further grouped by those who received placebo (staff, n=48; resident, n=14) or bamlanivimab (staff, n=51; resident, n=22) prior to vaccination (bottom) and (**B**) Binding antibody titers against spike-RBD-E484Q and spike-NTD are shown for samples from fully vaccinated participants who received SpikeVax (n=39) or Comirnaty (n=96) vaccine (top) and further grouped by those who received placebo (SpikeVax, n=21; Comirnaty, n=41) or bamlanivimab (SpikeVax, n=18; Comirnaty, n=55) prior to vaccination (bottom). Antibody titers were rescaled after adjusting for covariates. Boxes and horizontal bars denote the IQR and the median, respectively. Length of whiskers corresponds to 1.5 times the IQR. Statistical analysis was done using a linear model (two-sided test with α level of 0.05)

These binding antibody titer data were grouped into participants who were either staff or residents (Fig. 2A) and participants who received either SpikeVax or Comirnaty (Fig. 2B) to ascertain first whether the immune response to full vaccination differs between these two groups and secondly whether bamlanivimab infusion disparately affected these groups. Antibody titers from staff (median age 43 years) were 2.7 and 2.3 times (p<0.001) higher than antibody titers from residents (median age 72 years) against spike-RBD-E484Q and spike-NTD, respectively (Fig. 2A). The effect of bamlanivimab on vaccine-induced antibody titers against spike-RBD-E484Q and spike-NTD (p=0.388 and p=0.105, respectively) was similar for both residents and staff (Fig. 2A). There was no significant difference between antibody titers for participants who received SpikeVax or Comirnaty against either spike-RBD-E484Q or spike-NTD (p=0.722 and p=0.397, respectively) (Fig. 2B). For participants who received either vaccine, the effect of bamlanivimab on the vaccine-induced antibody titers against spike-RBD-E484Q and spike-NTD was also not significantly different (p=0.922 and p=0.756, respectively).

These same trends were also observed for antibody titers measured against the SARS-CoV-2 Beta variant (B.1.351), which is also not recognized by bamlanivimab (*11*). Compared with placebo, treatment with bamlanivimab resulted in a 1.7-fold lower titer against the Beta variant (fig. S1A). These antibody titer data were grouped into participants who were either staff or residents (fig. S1B) and participants who received either SpikeVax or Comirnaty (fig. S1C). Mirroring the results shown in Fig. 2A, antibody titers from staff were 2.9-fold (p<0.001) higher than titers from residents against the Beta variant (fig. S1B). The effect of bamlanivimab on vaccine-induced antibody titer against the Beta variant (p=0.326) was similar for both residents and staff. There was also no significant difference in antibody titers for participants who received either SpikeVax or Comirnaty against the Beta variant (p=0.842) (fig. S1C). For participants who received either vaccine, the effect of bamlanivimab on the vaccine-induced antibody titer against the beta variant was also not significantly different (p=0.77).

For completeness, antibody titers to the wildtype full-length spike and wildtype RBD (table S1) were also determined, even though bamlanivimab binds and neutralizes these variants (*7*). As expected, there was a strong correlation between bamlanivimab exposure and the measured titer against both bamlanivimab-binding antigens (ρ = 0.8 and ρ = 0.7 respectively, p<0.001). The infusion of bamlanivimab contributed to the measured longitudinal antibody titers compared with the endogenous antibody response to COVID-19 vaccination measured in participants who received placebo (fig. S2). There were no significant differences in antibody titers against the wildtype full-length spike (p=0.924) or wildtype RBD (p=0.363) for fully vaccinated participants who received either bamlanivimab or placebo (fig. S3).

Since the staff (n=99) included participants who were at high-risk of developing severe COVID-19, an additional analysis compared the immune responses of high-risk staff participants (n=45), with non-high-risk staff participants (n=54, fig. S4). Of these 45 high-risk staff participants, 13% were over 65 years old and at high-risk by pre-specified definition (*13*). The intention of this comparison was to ascertain, first, whether antibody titers following full vaccination differed between these risk groups and secondly, whether bamlanivimab infusion disparately affected these risk groups. Antibody titers for non-high-risk staff and high-risk staff were similar against both Spike-RBD-E484Q and spike-NTD (p=0.594 and p=0.348, respectively) (fig. S4). The effect of bamlanivimab on vaccine-induced antibody titer against Spike-RBD-E484Q resulted in a significantly lower titer (-1.8-fold, p=0.037) for high-risk staff compared with non-high-risk staff (fig. S4A). However, this effect was not observed against spike-NTD, where the effect of bamlanivimab was similar for the two groups (p=0.249) (fig. S4B).

### Anti-SARS-CoV-2 mAb infusion had minimal impact on potency of antibodies elicited by full COVID-19 vaccination.

**Fig. 3.**
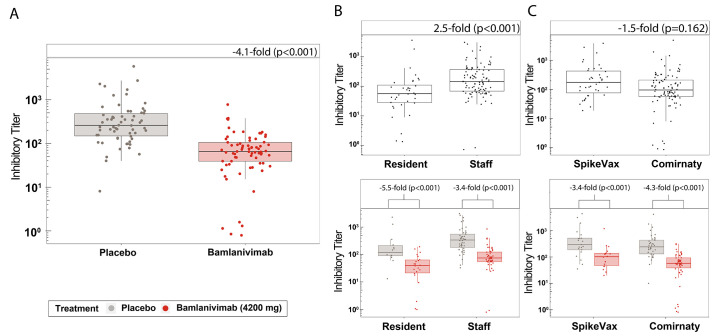
The minimal reduction in inhibitory potency of endogenous antibodies due to prior bamlanivimab infusion did not differ between staff and residents or SpikeVax or Comirnaty-treated patients. RBD**-**ACE2 binding inhibitory titers (IT) (1/IC_50_) are shown for serum samples collected from fully vaccinated participants (**A**) who had received placebo (n=62) or bamlanivimab (n=73) prior to vaccination, (**B**) who were resident (n=36) or staff (n=99) (top) and further grouped by those who received placebo (resident, n=14; staff, n=48) or bamlanivimab (resident, n=22; staff, n=51) (bottom), or (**C**) who received SpikeVax (n=39) or Comirnaty (n=96) (top) and further grouped by those who received placebo (SpikeVax, n=21; Comirnaty, n=41) or bamlanivimab (SpikeVax, n=18; Comirnaty, n=55) (bottom). Inhibition titers were measured as 1/IC_50_ and adjusted for covariates. Boxes and horizontal bars denote the IQR and the median reciprocal IC_50_, respectively. Length of whiskers corresponds to 1.5 times the IQR. Statistical analysis was done using a linear model (two-sided test with α level of 0.05).

The potency of the endogenous antibodies produced in response to full vaccination was evaluated in two ways: based on angiotensin-converting enzyme 2 (ACE2) binding inhibition measured with the custom multiplex assay and pseudovirus neutralization activity using a vesicular stomatitis (VSV)-based pseudovirus. Inhibition titers express how effectively the endogenous antibodies inhibited RBD-(E484Q)-ACE2 binding. Compared with placebo, receipt of bamlanivimab resulted in a 4.1-fold (p<0.001) lowering in ability of the endogenous antibody response to inhibit ACE2 binding (Fig. 3A). These inhibition titer data were grouped into participants who were either staff or residents (Fig. 3B) and participants who received either SpikeVax or Comirnaty (Fig. 3C), to ascertain first, whether the potency of antibodies elicited following full vaccination differs between these groups and secondly, whether bamlanivimab infusion prior to full vaccination disparately affects these groups.

The inhibition titers of endogenous antibodies were measured as 2.5 times (p<0.001) higher for staff than for residents (Fig. 3B). There was no disparity in inhibition titers between participants who received SpikeVax or Comirnaty (p=0.162) (Fig. 3C). The magnitude of the bamlanivimab effect on inhibition titers was similar for both resident and staff (p=0.233) (Fig. 3B) and for participants who had SpikeVax or Comirnaty (p=0.574) (Fig. 3C). We observed no difference in inhibition titers between non-high-risk and high-risk staff (p=0.441), nor was the magnitude of the bamlanivimab effect on inhibition titers different for these groups (p=0.084) (fig. S5A).

**Fig. 4.**
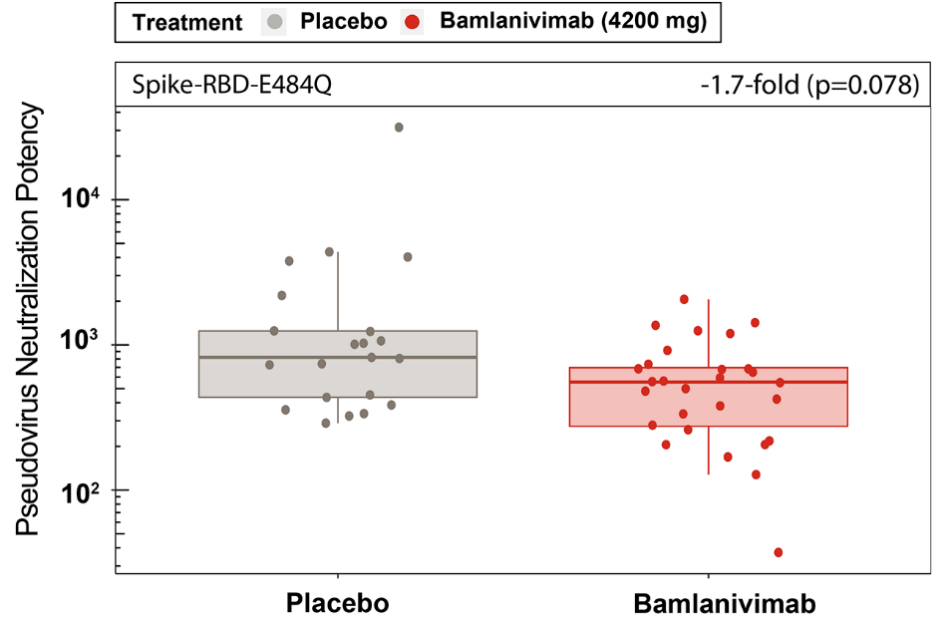
**Pseudovirus neutralization potency against spike-E484Q did not differ between placebo- and bamlanivimab-treated individuals**. Pseudovirus neutralization potency against spike-E484Q are shown for serum samples collected from 49 fully vaccinated participants who had received placebo (n=21) or bamlanivimab (n=28) prior to full vaccination. Pseudovirus neutralization potency measured as 1/NT_50_ and adjusted for T1 and T2 covariates. Boxes and horizontal bars denote the IQR and the median reciprocal NT_50_, respectively. Length of whiskers corresponds to 1.5 times the IQR. Statistical analysis was done using a linear model (two-sided test with α level of 0.05).

To assess the functional polyclonal antibody response against the full-length spike, neutralization potency was measured using a VSV-based pseudovirus for samples from a subset of participants (n=49; 21 placebo, 28 bamlanivimab). The participants sampled had all received their first vaccine dose 64 days or fewer (median 57 days) after a bamlanivimab or placebo infusion and thereby were the most likely to exhibit an effect of bamlanivimab infusion on the immune response to subsequent vaccination. There was no statistically significant difference in pseudovirus neutralization potency against spike-E484Q for participants who received either placebo or bamlanivimab (p=0.078) (Fig. 4).

We observed no difference in neutralization potency against spike-E484Q pseudovirus between non-high-risk and high-risk staff (p=0.34), and the magnitude of the bamlanivimab effect was also similar for non-high-risk and high-risk staff against spike-E484Q (p=0.085, fig. S5B). There was also no statistically significant difference (-2.0-fold, p=0.271) in the magnitude of the effect of bamlanivimab on the pseudovirus neutralization potency against spike-E484Q for participants, whether they were staff or resident (fig. S5C). To corroborate the results against spike-E484Q pseudovirus, the neutralization potency was also evaluated against the Beta variant (B.1.351) pseudovirus for the same subset of patients and similarly there was no statistically significant difference (-1.2-fold, p=0.465) in the effect of bamlanivimab on antibody potency compared with placebo (fig. S6A). Furthermore, there was a significant strong Spearman correlation, ρ, of 0.8 (p<0.001) between the neutralization potency data against spike-E484Q and the Beta variant pseudoviruses (fig. S6B). These strong correlations also extended to pseudovirus data from participants who received either bamlanivimab or placebo (fig. S6B).

### Antibody binding titers, ACE2-RBD binding inhibition titers, and pseudovirus neutralization results show a high degree of correlation.

**Fig. 5.**
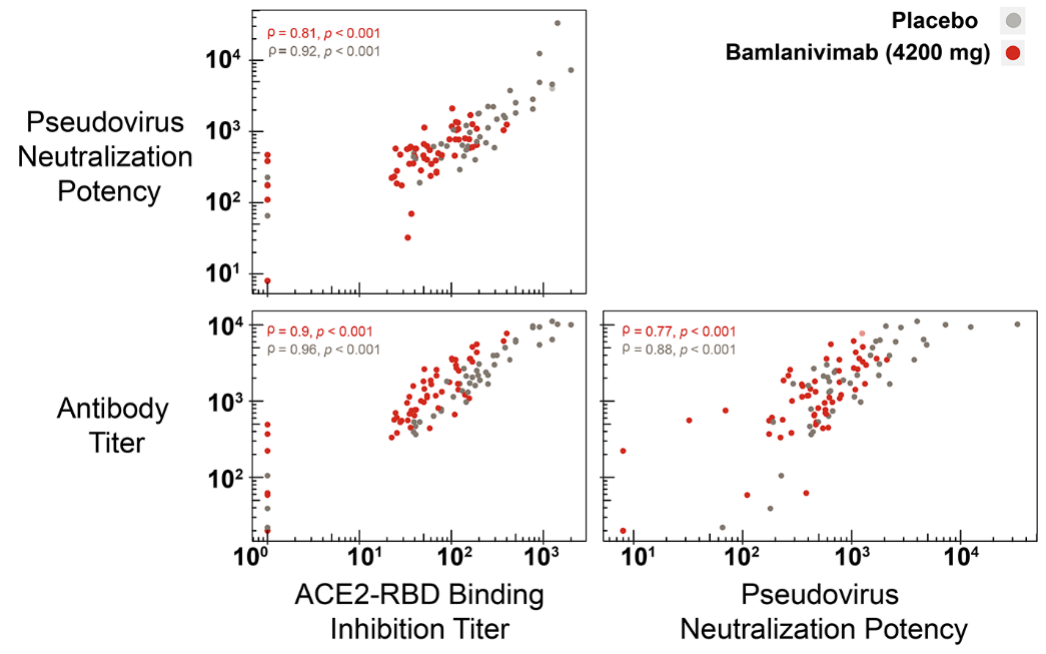
A strong correlation between results from three different assays indicate minimal impact of prior bamlanivimab infusion on endogenous antibody response to full COVID-19 vaccination. Correlation matrices showing the degree of correlation between paired results from ACE2-RBD binding inhibition assays, pseudovirus neutralization assays (spike-E484Q), and antibody titers (spike-RBD-E484Q). Correlation strength was determined for participants who received placebo (n=62) or bamlanivimab (n=73) using cor.test() function in R with asymptotic *t* approximation to calculate the Spearman correlation. ρ represents the Spearman correlation; p represents the p-value.

To corroborate the results obtained using different assays to measure antibody titers, inhibition titers, and pseudoviral neutralization potency, the correlation strength was determined between all assay results against Spike-RBD-E484Q for the subset of 114 samples from 49 participants. This correlation analysis was limited to the sample of 49 participants for whom the pseudovirus neutralization assay was completed. A high degree of correlation was observed between all assay results (Fig. 5). The Spearman correlation, ρ, was determined as 0.87 (p<0.001) for ACE2-RBD binding inhibition titer data with pseudovirus neutralization data. Similarly, significant correlations were observed between pseudovirus neutralization data and antibody titers (ρ =0.84, p<0.001) and between ACE2-RBD binding inhibition titers and antibody titers (ρ =0.91, p<0.001). These strong correlations also extended to data from participants who received either bamlanivimab or placebo (Fig. 5).

### Robust longitudinal antibody responses to COVID-19 vaccine were observed irrespective of serum bamlanivimab concentration at time of vaccination.

**Fig. 6.**
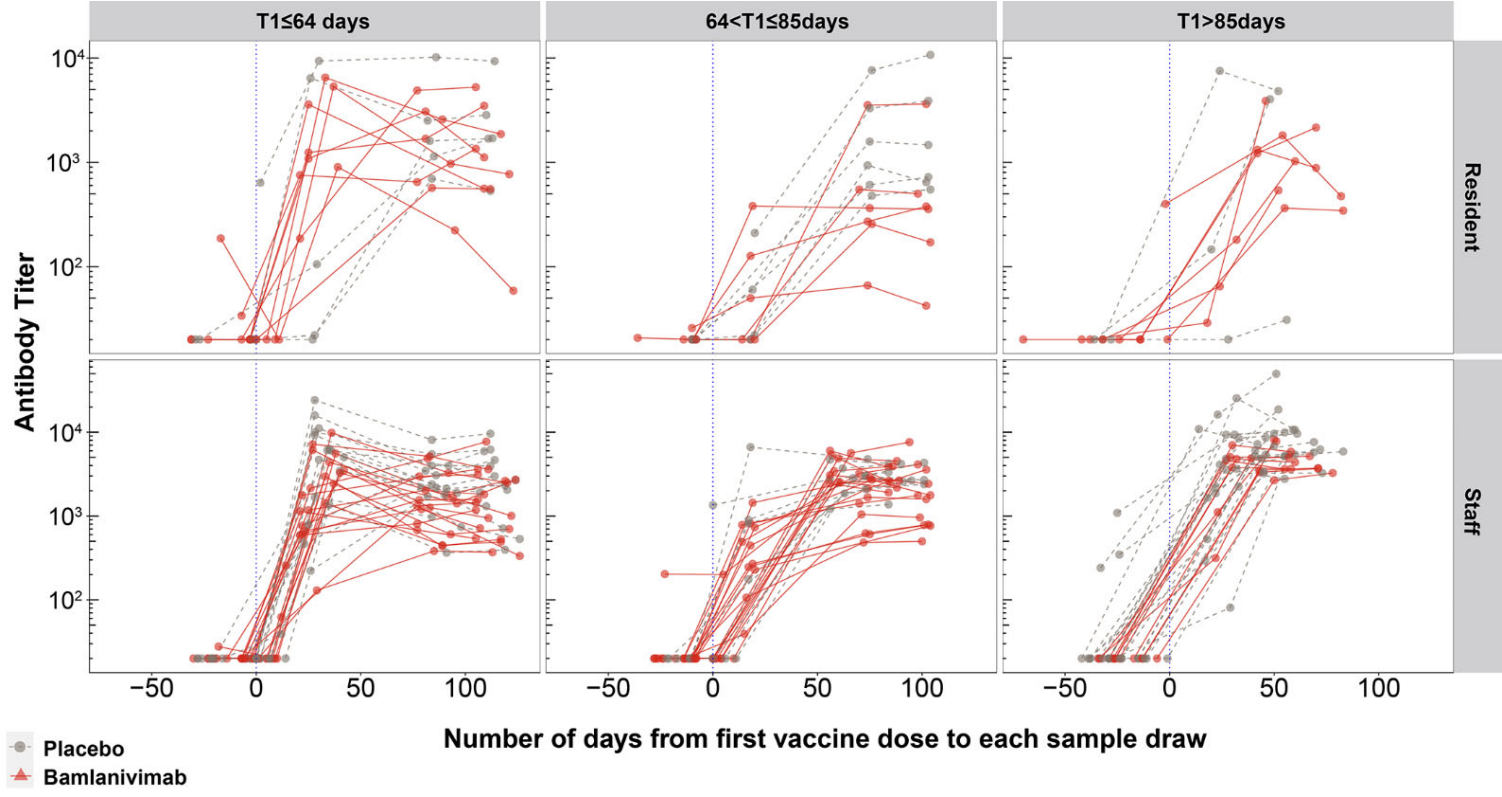
Robust endogenous antibody responses were elicited to the first COVID-19 vaccine dose, irrespective of duration since prior bamlanivimab infusion. Longitudinal binding antibody responses against spike-RBD-E484Q were arranged into three groups based on tertiles of the interval (in days) between bamlanivimab or placebo infusion and first vaccine dose, T1. The three columns (left to right) correspond to T1≤64 days, 64<T1≤85 days, and T1>85 days (n=50, 42 and 43 participants) respectively. The vertical blue dotted line denotes the timepoint where participants receive the first dose of vaccine. Each line connects sample titers from a single participant. The top row shows antibody titers of participants who were residents, and the bottom row represents antibody titers of participants who were staff.

To visualize the longitudinal antibody responses to the COVID-19 vaccine, antibody titers measured from all samples (n=498) from 135 participants were evaluated against spike-RBD-E484Q (Fig. 6). Since there was temporal variability in the number of days between receiving bamlanivimab or placebo and the first vaccine dose (from 43 to 127 days) the participants were divided into three groups based on the interval (T1) between bamlanivimab or placebo infusion and first vaccine dose. The longitudinal representation of the antibody titers against spike-RBD-E484Q depicts the antibody response after the first COVID-19 vaccine dose for all fully vaccinated participants, whether infused with bamlanivimab or placebo (Fig. 6). The three groups were T1≤64 days, 64 days<T1≤85 days and T1>85 days with 50, 42 and 43 participants respectively, where 64 days and 85 days are the tertiles of T1.

**Fig. 7.**
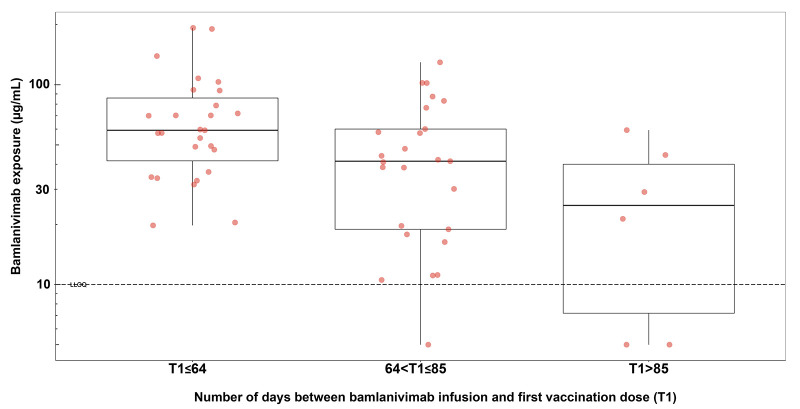
Bamlanivimab serum concentration is contingent on the number of days since bamlanivimab infusion. Bamlanivimab serum concentration (in μg/mL) at time of first COVID-19 vaccination dose is shown for participants arranged into three groups based on the interval (in days) between bamlanivimab infusion and first vaccine dose, T1. The three groups are T1≤64 days, 64<T1≤85 days, and T1>85 days (n=50, 42 and 43 participants, respectively). Each data point represents a single participant with samples collected ±14 days of the first vaccination dose. Where a participant had more than one qualifying sample, the geometric mean was determined. Boxes and horizontal bars denote the IQR and the median bamlanivimab serum concentration, respectively. Length of whiskers corresponds to 1.5 times the IQR. The dashed black line represents the lower limit of quantification for assay (LLOQ = 10 μg/mL).

For the participants in these three groups who were assigned 4200 mg of bamlanivimab, pharmacokinetic data were analyzed to determine their serum bamlanivimab concentration at the time of first vaccination dose (Fig. 7). At the time of first vaccination dose (±14 days), the median bamlanivimab concentration was determined for participants in the three groups (T1≤64 days, 64<T1≤85 days and T1>85 days) as 59.2 μg/mL, 41.4 μg/mL and 25.2 μg/mL, respectively (Fig. 7). Despite differing bamlanivimab concentrations at the time of vaccination for participants included in each group (Fig. 7), there were no obvious differences between the immune responses of the three groups (Fig. 6). Further, the majority of participants across the three groups also exceeded the in vitro estimated 90% inhibitory concentration (IC_90_) (4.1 μg/mL) for bamlanivimab that is needed for protection from COVID-19 (*30*). Although the study did not allow for conclusions to be drawn about the immune response to the first vaccine dose, the data demonstrate that at no point in time did the groups divert beyond the small differences in titers observed at full vaccination. Similar results were also observed for longitudinal antibody titers against the spike-NTD (fig. S7).

## DISCUSSION

In this post-hoc analysis of the BLAZE-2 nursing home study, all participants demonstrated a robust immune response to full COVID-19 vaccination, regardless of preceding bamlanivimab or placebo infusion and irrespective of age, risk-category, and vaccine type. Furthermore¸ the interval between mAb infusion and COVID-19 vaccination did not affect this conclusion. These are important findings in the pursuit of informed treatment planning, particularly for individuals at high risk for severe disease, and support earlier COVID-19 vaccination for individuals who are currently deferring following mAb receipt, as per current CDC and WHO guidelines (*16*, *17*). Here, we demonstrate that SARS-CoV-2-naïve individuals who have received a prophylactic mAb can still mount a robust immune response to COVID-19 vaccination; therefore, the benefit of prompt COVID-19 vaccination outweighs the minimal effect of a prior prophylactic COVID-19 mAb.

The immune response of participants to COVID-19 vaccination was evaluated using assays to measure the antibody titers, ACE2-RBD binding inhibition titers and pseudoviral neutralization. There was a high degree of correlation between all assay results, suggesting reliable trends that demonstrated minimal differences in immune response to COVID-19 vaccination for participants who previously received either bamlanivimab or placebo. Importantly, assays were measured against multiple domains of the spike protein that bamlanivimab does not efficiently bind to, thereby reflecting the endogenous antibody response and with consistently strong correlations.

With respect to the vaccine-induced antibody potency evaluated using two different assays, there was no difference in pseudovirus neutralization potency between individuals who received either bamlanivimab or placebo, though lower inhibition titers were detected. The contrast in measured antibody potency is likely explained by the breadth of epitopes assessed in each assay; the ACE2-RBD binding inhibition assay assesses RBD-binding antibodies only, whereas the pseudovirus assay assesses the functionality of the polyclonal antibody response against the full-length spike. The strong correlation between the potency assays and the preservation of high concentrations of neutralizing activity suggests minimal impact on immune protection conferred by COVID-19 vaccination, regardless of prior bamlanivimab or placebo infusion. Previous studies investigating immune evasion have determined that complete loss of antibody neutralizing activity against SARS-CoV-2 variants corresponded to a greater than 40-fold change reduction compared with neutralizing activity against wildtype pseudovirus (*31*, *32*). Therefore, despite some decrements in antibody potency in participants who received prior bamlanivimab infusion compared with placebo, the magnitudes of these reductions were still within range with studies on correlates of protection and therefore may not translate to relevant clinical interference.

As many researchers endeavor to identify immune correlates of protection, growing evidence indicates that binding and neutralizing antibodies correlate with COVID-19 vaccine efficacy (*4*, *18*, *19*). A recent modelling study identified a strong non-linear relationship between mean neutralization titers and the reported protection of vaccines and predicted that the 50% protective neutralization titers of a COVID-19 vaccine was achieved at approximately 20% of mean convalescent titers (*33*). Another study also estimated vaccine efficacy based on antibody marker titers and showed that a 10-fold lower antibody titer for SpikeVax vaccine recipients only reduced vaccine efficacy from 96.1% to 90.7% (*18*). In our study, antibody titers for fully vaccinated participants who had previously received bamlanivimab compared with placebo were reduced by two-fold or less. Therefore, despite some differences, the magnitude of these decrements was minimal when compared with studies on correlates of protection. Higher antibody titers in individuals who were fully vaccinated with SpikeVax compared with Comirnaty have been reported (*34*), yet each vaccine has demonstrated more than 90% efficacy in preventing COVID-19 illness (*35*, *36*). We concluded here that there was no difference in the antibody response to different mRNA COVID-19 vaccines and that bamlanivimab infusion also did not disparately affect participants who had either vaccine.

Understanding the endogenous antibody responses to COVID-19 vaccines is particularly important for older and immunocompromised individuals, who are at high-risk of developing severe COVID-19 and have therefore been the targeted recipients of prophylaxis or treatment with COVID-19 mAbs (*37*–*40*). The participants in this study included both residents and staff of U.S. skilled nursing and assisted living facilities, allowing us to evaluate the potential impact of age and risk categorization on the vaccine-induced antibody response following receipt of prophylactic bamlanivimab or placebo. First, staff (median age 43 years) were shown to have significantly (p<0.001) higher antibody titers and greater antibody inhibition potency than residents (median age 72 years), which is consistent with the literature that has shown stronger immune responses in younger individuals (*41*). Secondly, bamlanivimab had a similar effect on the antibody titers and potency in both residents and staff, which informs that bamlanivimab did not disparately affect the immune response to full COVID-19 vaccination of participants of different ages. An additional sub-analysis was completed for the staff who were dichotomized into non-high-risk and high-risk participants based on pre-determined risk factors for developing severe COVID-19 (*13*). With the exception of the titer against Spike-RBD-E484Q (p=0.037), no differences in antibody titer or potency were observed between the two risk groups, although a trend toward lower titers was observed for all measures in high-risk staff. Overall, these findings align with the observed differences between residents and staff, suggesting that the aggregate of risk factors influences the overall response. These results therefore demonstrate that participants can mount a strong immune response to COVID-19 vaccination following a mAb infusion, irrespective of age and high-risk categorization.

This is an important finding as therapeutic mAbs have been an important part of the treatment armamentarium for individuals who are at high risk of developing severe COVID-19, and the current lack of data resulted in uncertainty with regard to defining the best therapeutic guidance for this group. Although this post-hoc analysis evaluated the immune response of mAb infused individuals to two doses of a COVID-19 vaccine, a further consideration is that additional vaccine doses (boosters) are now part of the protection paradigm and should therefore also be considered in the context of previous mAb administration. Based on the reduced protective efficacy of COVID-19 vaccines over time and against certain SARS-CoV-2 variants (*42*, *43*), additional doses are increasingly administered to boost the immune response, with high-risk individuals being prioritized (*44*). This post-hoc analysis concluded that individuals who have previously received a COVID-19 mAb can proceed with COVID-19 vaccination and mount a strong immune response, which could be further boosted by additional vaccine doses.

The continuous evolution of SARS-CoV-2 has led to the emergence of variants of concern that have challenged the efficacy and protective longevity of some COVID-19 mAbs and vaccines. For example, the emergence of the SARS-CoV-2 B.1.1.529 (Omicron) variant is a major development in the field of COVID-19 vaccines and therapeutics. Reports have shown a reduction in neutralization titers against Omicron compared with wildtype for those fully vaccinated with either Comirnaty or SpikeVax (*42*, *43*). However, a third booster dose of either vaccine elicits a potent antibody response against Omicron, which has prompted the CDC to strengthen their recommendation on booster doses (*43*, *45*–*47*). The current study did not specifically include Omicron; this variant has been shown to evade neutralization by bamlanivimab and, in this way, is analogous to the three SARS-CoV-2 proteins used in this study (*48*–*50*). The significant correlation (p<0.001) between neutralization potencies against the Beta variant and spike-E484Q pseudoviruses demonstrated that the effect of prior bamlanivimab infusion was comparable against different SARS-CoV-2 proteins to which bamlanivimab does not bind. As such, we posit similar reductions in titers and neutralization potency due to preceding bamlanivimab infusion against Omicron or any other variant that bamlanivimab does not bind to effectively.

An important consideration for this post-hoc analysis is understanding bamlanivimab concentration in patients at the time of vaccination. An earlier pharmacokinetics (PK) modelling study determined that there is a linear relationship between bamlanivimab dose and exposure and that the half-life of bamlanivimab is approximately 17 days (*51*). These three groups thereby represented participants with different serum concentrations of bamlanivimab at the time of first vaccination dose (59.2 μg/mL, 41.5 μg/mL and 25.2 μg/mL). Despite differing serum bamlanivimab concentrations at the time of vaccination for participants in each of the three groups, no discernible differences in antibody titers in response to the vaccine were observed. As such, we hypothesize similar findings for other mAbs, regardless of how exposure is achieved (for example, through half-life extension or high initial dose). Previous population PK analyses have also shown that there is no difference in the PK of bamlanivimab in geriatric patients compared to younger patients (*9*, *30*). The bamlanivimab dose (4200 mg) administered in this trial was higher than the bamlanivimab dose used in clinical practice (700 mg). As such, most participants far exceeded the in vitro estimated IC90 (4.1 μg/mL) at the time of vaccination (*30*). Therapeutic mAbs for COVID-19 are known to reduce viral load (*7*, *52*) and we have previously shown that high viral load is associated with high antibody titers (*53*). Therefore, there was the potential for bamlanivimab to markedly reduce the endogenous immune response to COVID-19 vaccination. However, similar to previous findings (*53*), any reduction in antigen load due to therapeutic mAbs was not sufficient to severely blunt the immune response, in this case to COVID-19 vaccination.

Although this study did not allow for conclusions to be drawn about the immune response to the first dose of vaccine, the longitudinal titer data did not deviate beyond the small difference in titer observed at full vaccination. There were also no obvious differences in the antibody responses between the three groups, despite varying serum bamlanivimab concentrations at the time of vaccination. These data also included 97 participants (70%) who had received the first vaccine dose within 90 days of bamlanivimab or placebo infusion (*16*, *17*).

Given the retrospective nature of this study, there are inherent limitations with respect to study design. For example, the post-hoc analysis population was determined as described in the Materials and Methods, thereby limiting the sample size and demographics. The vaccine type and timing were also variable and determined by circumstance. A total of 498 samples from fully vaccinated participants who met the post-hoc analysis inclusion criteria were assessed for antibody titer and ACE2 binding inhibition titers using a custom assay. Owing to the custom nature of this assay, it was decided to perform a standard pseudovirus neutralization assay to complement and corroborate these data. Due to logistical limitations, purposive sampling was used to select samples for the pseudovirus assay from a subset of participants (n=49) who received their first vaccine within 64 days of either bamlanivimab or placebo. This group of participants were selected as they had the highest serum bamlanivimab concentration at time of vaccination, thus representing those most likely to exhibit an effect of bamlanivimab on pseudovirus neutralization potency. Despite the smaller sample, the neutralization potency against spike-RBD-E484Q and the Beta variant pseudoviruses were strongly correlated, further supporting our interpretation of minimal impact. This post-hoc analysis only assessed the impact of a single mAb on the endogenous immune response to a COVID-19 vaccine. However, we hypothesize similar results for other mAbs that reduce viral load upon administration (*52*). This study was also limited to participants who received an mRNA vaccine; therefore, it is not known whether these findings extend to other vaccine types. This analysis also did not assess the simultaneous administration of vaccine and mAb; however we have recently shown that patients with mild or moderate COVID-19 that were administered bamlanivimab or bamlanivimab and etesevimab together early in infection elicited a wide breadth of antigenic responses to SARS-CoV-2 (*53*).

In conclusion, this post-hoc analysis expands the current understanding of the impact of receiving a prophylactic monoclonal antibody infusion, along with other factors, on the endogenous immune response to full COVID-19 vaccination. There was a high degree of correlation between all assay results of vaccine-induced antibody titer and potency against different SARS-CoV-2 proteins supporting the conclusion that participants mount a strong immune response to full COVID-19 vaccination, regardless of preceding prophylactic mAb infusion and irrespective of age, risk-category and vaccine type. Despite some decrements in antibody titers and potency for fully vaccinated participants who had previously received bamlanivimab compared with placebo, these differences were minimal when compared with the current literature. With small reductions in antibody concentrations and no difference in neutralization activity, the clinical impact of antecedent bamlanivimab infusion appears limited. These findings are pertinent for informing public health policy, particularly for SARS-CoV-2-naïve individuals and those at high-risk of developing severe COVID-19, who can receive immediate protective immunity from COVID-19 mAbs while awaiting the development of durable polyclonal vaccine-induced protection. These results demonstrate that the benefit of receiving a COVID-19 vaccination at the earliest opportunity outweighs any minimal effect on the endogenous immune response due to prior COVID-19 mAb infusion.

## MATERIALS AND METHODS

### Study Design

The BLAZE-2 trial was a phase 3, randomized, double-blind, placebo-controlled, single-dose SARS-CoV-2 prevention study and has been described previously (*22*). Participants of the BLAZE-2 study included residents and staff of U.S. skilled nursing and assisted living facilities who were randomized to the study drug, bamlanivimab (4200 mg) or placebo. As per the trial protocol, serum samples were collected from participants at baseline (prior to bamlanivimab or placebo infusion) and post-baseline samples were collected at day 29, day 57, day 85, day 141, and day 169 (*22*). All donors provided written informed consent for the use of blood and blood components (such as peripheral blood mononuclear cells, serum or plasma). The BLAZE-2 trial was initiated with the objective of evaluating prophylactic efficacy of bamlanivimab in individuals at high-risk of developing severe COVID-19 disease. In an unscheduled component of this study, these high-risk individuals were selected as part of the U.S. vaccination program to be amongst the first to receive two COVID-19 mRNA vaccine doses (Comirnaty or SpikeVax). At the time of receipt, the Comirnaty and SpikeVax mRNA vaccines had been granted Emergency Use Authorizations (EUAs) by the U.S. FDA (*36*, *54*).

The selection process for participant inclusion in this post-hoc analysis is presented in fig. S8. The participant sample size for this post-hoc analysis was dictated by circumstance and included BLAZE-2 participants who met all of the following criteria: (a) participants were in the prevention cohort of the BLAZE-2 trial; (b) participants tested negative for SARS-CoV-2 throughout the study, as determined using both reverse transcription polymerase chain reaction (RT-PCR) assessed at baseline and then weekly until day 57 and on days 85 and 141 and nucleocapsid (NCP) antibody assay (Cobas, Roche Diagnostics) assessed on days 1, 29, 57, 85, and 141); (c) participants had received two COVID-19 vaccine doses subsequent to a bamlanivimab or placebo infusion; and (d) participants had at least one serum sample obtained more than 2 weeks following the second vaccine dose. The CDC describes an individual as fully vaccinated after 2 weeks following the second COVID-19 vaccine doses in a 2-dose series, such as for Comirnaty or SpikeVax (*23*). The baseline characteristics for the 135 participants who met these criteria and were included in this post-hoc analysis are presented in Table 1.

Participants received COVID-19 vaccines (SpikeVax or Comirnaty) when they were offered to the respective nursing and assisted living facilities by the U.S. government. Consequently, participants received a first vaccine dose at different timepoints (starting at day 44 onwards) following bamlanivimab or placebo infusion. Participants received the second vaccine dose following the recommended period specified in the EUA factsheet for each vaccine (21 and 28 days later for Comirnaty and SpikeVax, respectively) (*24*, *25*).

To evaluate the effect of bamlanivimab infusion on the subsequent antibody response to full COVID-19 vaccination, the following criteria were adopted to select samples from the 135 participants for statistical analysis: (a) exclusion of participant samples that were obtained prior to the receipt of the second vaccine dose; (b) exclusion of samples from participants who did not have a record of a second vaccine dose at the time of the analysis; (c) exclusion of samples which were obtained within 14 days of the second vaccine dose (samples were only used if collected after full vaccination, as determined by the CDC) (*23*); and (d) if more than one sample was obtained after a participant was fully vaccinated, the lattermost sample was selected for analysis. The serum sampling period was pre-specified in the BLAZE-2 protocol and the final serum samples were collected on day 169 (*22*). A total of 498 samples met these criteria, but one sample was excluded due to a sample handling error. A total of 498 samples from 135 participants were included in this post-hoc analysis and assays were performed to measure antibody titers and ACE2-RBD binding inhibition titers. Prior to use in each assay, serum samples were centrifuged for 5 min at 10000 x *g* to pellet any debris.

Since the ACE2-RBD binding inhibition titer results were collected using a custom Luminex-based assay, a standard VSV pseudoviral assay was also performed to complement and corroborate this data. Owing to the substantial number of serum samples collected, the pseudoviral assay was performed on a purposive sample of 74 samples from 49 fully vaccinated participants (Fig. 6).

Longitudinal analysis of antibody titers measured from all 498 samples obtained from the 135 participants facilitated the visualization of the antibody response by each individual to COVID-19 vaccination. Since the timing of vaccine dosing varied for each individual, the participants were organized into three groups based on the interval between receipt of bamlanivimab or placebo and the subsequent receipt of the first COVID-19 vaccination dose, T1. Each of the 135 participants were placed into one of the three groups: T1≤64 days, 64<T1≤85 days, and T1>85 days.

### Luminex-Based Assay

Luminex xMAP technology is an established, multiplex, flow cytometry-based platform that allows the simultaneous quantitation of many protein analytes in a single reaction (*55*). A custom Luminex-based assay was developed to measure serology and antibody ACE2-RBD binding inhibition in a single assay. Antigen-coated microspheres were used to detect and quantitate endogenous antibodies against SARS-CoV-2 proteins, including spike-NTD and several RBD epitopes (table S1), to which bamlanivimab does not efficiently bind (*7*, *56*).

Patient serum samples were titrated (1:20 to 1:4.3x10^8^) as a single dilution curve in phosphate buffered saline-high salt solution (PBS-HS; 0.01 M PBS, 1% bovine serum albumin [BSA], 0.02% Tween, 300 mM NaCl). Diluted serum samples were combined with Luminex MAGPlex microspheres coupled with individual antigens and a recombinant, labeled RBD-phycoerythrin (PE) protein and incubated for 60 min to allow endogenous antibodies to bind to either the recombinant RBD-PE or to the antigen-coated Luminex beads. The solution was placed on a magnet, collecting the MAGPlex beads, and the supernatant was transferred to a new plate. The transferred solution was combined with ACE2-coated beads and incubated for 60 min, and the remaining beads were washed and incubated for 60 min with anti-IgG-PE beads to detect bound antibodies.

All the beads on both plates were then washed and resuspended in a PBS-1% bovine serum albumin (BSA) solution and read using a Luminex FlexMAP 3D System with xPONENT Software. Titers were determined from the median fluorescence intensity (MFI); the ability of the endogenous antibodies to inhibit RBD-ACE2 binding was calculated based on the half maximal inhibitory concentration (IC_50_) which represents the antibody titer where the ACE2-RBD binding is reduced by half. ACE2-RBD binding inhibition titers were assessed using the inverse of IC_50_.

### Pseudovirus production and characterization

E484Q mutagenesis reactions were performed using the QuickChange Lightning Site-Directed Mutagenesis Kit (Agilent #210519) using a template of a spike mammalian expression vector based on the Wuhan sequence (GenBank MN908947.3) with a deletion of the C-terminal 19 amino acids. For the Beta variant (B.1.351) pseudovirus, a consensus sequence representative of the Beta lineage was synthesized and incorporated by Gibson cloning. Pseudoviruses bearing mutant spike proteins were produced using the delta-G-luciferase recombinant Vesicular Stomatitis Virus (rVSV) system (KeraFast EH1025-p.m., Whitt 2010). Briefly, 293T cells were transfected with individual mutant spike expression plasmids, and 16 to 20 hours later, transfected cells were infected with VSV-G-pseudotyped delta-G luciferase rVSV. After 16 to 20 hours, conditioned culture medium was harvested, clarified by centrifugation at 1320 g for 10 min at 4°C, aliquoted and stored frozen at -80°C. Relative luciferase reporter signal read-out was determined by luciferase assay (Promega E2650) of extracts from VeroE6 cells infected with serially-diluted virus. Luciferase activity was measured on a PerkinElmer EnVision 2104 Multilabel Reader.

### Pseudovirus neutralization assays

Neutralization assays were carried out essentially as described previously (*57*, *58*). Serum antibodies were diluted 4-fold in assay media and 10-point, 3-fold titrations in 25% assay media were performed in 384-well polystyrene plates in duplicate using a Beckman (Biomek i5) liquid handler. Positive and negative control antibodies and an unrelated control (hIgG1 isotype) were tested in a 10-point, 3-fold serial dilution starting at 8 μg/mL, 2 μg/mL and 8 μg/mL, respectively, in 25% assay media. An empirically pre-determined fixed amount of pseudovirus expressing spike-RBD-E484Q or the Beta variant (B.1.351) spike (table S1) was dispensed by WDII liquid dispenser on titrated serum antibodies and controls and pre-incubated for 20 min at 37°C. Following pre-incubation, virus-antibody complexes were transferred by Biomek i5 to VeroE6 cells at a concentration of 8,000 cells per well in white, opaque, tissue culture treated 384W plates. Samples were then incubated for 16 to 20 hours at 37°C. Control wells included virus only (no antibody; 14 replicates) and cells only (14 replicates). Following infection, cells were lysed with Promega BrightGlo and luciferase activity was measured on the Biotek Synergy Neo2 Multimode Reader. Antibody neutralization potency was assessed using the inverse of NT_50_, defined as the antibody concentration, at which the viral replication has been reduced by 50% relative to the absence of antibodies.

### Pharmacokinetics Analysis

Serum concentrations of bamlanivimab were determined using a validated hybrid liquid chromatography-tandem accurate mass spectrometry method (*30*). Bamlanivimab concentrations were evaluated from 274 samples collected from all of the 73 bamlanivimab-infused participants. To evaluate serum bamlanivimab concentrations at the time of first vaccination dose, samples that were collected within 14 days before or after first vaccination dose were analyzed. Bamlanivimab concentration was therefore determined for 58 out of the possible 73 participants who received bamlanivimab, as not all participants had a sample draw within the specified timeframe. In each group, there were 27 participants (T1≤64 days), 25 participants (64<T1≤85 days) and 6 participants (T1>85 days). Where participants had two samples within ±14 days of vaccine dose, the geometric mean of these data was determined. For samples that did not reach the lower limit of quantification (LLOQ), these concentrations were imputed as LLOQ/2, where LLOQ = 10μg/mL.

### Statistical Analysis

For the serial dilution-based serology assay, titers could either be defined as the reciprocal of the highest dilution of the sample above a pre-determined “cut point” value or be derived based on interpolating assay values that straddle the “cut point” (*59*). The latter method was used in the forementioned serology assay. The serology titer data were evaluated on a log base 10 scale.

To calculate IC_50_ of data from the ACE2-RBD binding inhibition component of the serology assay, a 4-parameter logistic function was used to estimate the absolute IC_50_ based on 1/dilution factor. To calculate NT_50_ of data from the pseudovirus neutralization assay, a 4-parameter logistic function was used to estimate the absolute NT_50_ based on 1/dilution factor (bottom is fixed at 0 for pseudovirus NT_50_). For the pseudovirus neutralization assay, if a sample indicates no neutralization or has a poor fit (the standard error of the NT_50_ is not estimable or the estimated NT_50_ is larger than the maximum 1/dilution factor), the NT_50_ value was imputed as 0.125 (twice the maximum 1/dilution factor). For the ACE2-RBD binding inhibition assay, if a sample indicated no inhibition, the IC_50_ was imputed to 1 (20 times the maximum 1/dilution factor). The bamlanivimab effect (compared with placebo) was evaluated based on log_10_ scale of 1/IC_50_ for RBD-ACE2 binding inhibition potency or of 1/NT_50_ for pseudovirus neutralization potency.

Since COVID-19 vaccination was not a planned component of this study, the temporal variability in vaccine dosing for each participant relative to the sampling schedule had to be accounted for in the analysis. For each participant, let T1 denote the interval (days) between bamlanivimab or placebo infusion and first COVID-19 vaccine dose and T2 denote the interval (days) from second COVID-19 vaccine dose to each sampling visit. T1 and T2 were included as covariates in the linear model used for hypothesis testing (two-sided test with α level of 0.05).

To investigate the effects of different variables of interests, such as treatment group (bamlanivimab or placebo), patient group (resident or staff), vaccine type (Comirnaty or SpikeVax), or risk group in staff patients (high risk versus non-high risk), the relevant variable was also included in the linear model. To compare the effect of bamlanivimab or placebo infusion on these different patient groups, the interaction of treatment group (bamlanivimab or placebo) x selected variable and corresponding selected variable were also included in the linear model. Adjustments for multiple testing were not conducted. The statistical analyses were performed with R software (version 4.0.3) (*60*).

To visualize the results after adjusting for T1 and T2, residuals adjusted for T1 and T2 (Eq. 1) were rescaled to either titer or 1/IC_50_ or 1/NT_50_ scales, depending on which response variable was evaluated in the linear model, where Y = either log_10_(titer) or log_10_(1/IC_50_), or log_10_(1/NT_50_). Let Y* denote the rescaled Y. Since Y ~ N(μ_Y,_ σ_Y_^2^_)_ and e  ~ N(μ_ε,_ σ_ε_^2^_),_ ${\hat{Y}}^{*}=\;\left|{\hat{µ}}_{Y}\right.+\;\frac{\hat{e}-{\hat{µ}}_{e}}{{\hat{\sigma }}_{e}}{\hat{\sigma }}_{Y}$ _,_ where the "hat ” indicates the estimated value.


Eq. 1 $$Y_{i}=T1_{i}\;+\;T2_{i}\;+{\text{e}}_{i},\;where\;i\;=\;1,\;...,\;n$$

The minimum significant ratio (MSR) is a statistical parameter used to measure assay variability (*61*). The replicate experiment MSR was determined as described in (*62*) for the ACE2-RBD binding inhibition assay and pseudovirus assay used in this post-hoc analysis. Pseudovirus assay exhibits higher variability (MSR ≈ 5) compared to ACE2-RBD binding inhibition assay (MSR ≈ 1.2), which reflects the breadth of the epitope assessed in each assay. The ACE2-RBD binding inhibition assay has greater precision, as it assesses RBD-binding antibodies only, whereas the pseudovirus assay assesses the functionality of the polyclonal antibody response against the full-length spike.

## Supplementary Materials

### Other Supplementary Material for this manuscript includes the following:

MDAR Reproducibility Checklist
