# Supplementary material for: The anti-SARS-CoV-2 monoclonal antibody, bamlanivimab, minimally impacts the endogenous immune response to COVID-19 vaccination
Source: Sci Transl Med. 2022 Jun 9:eabn3041. doi: 10.1126/scitranslmed.abn3041 (PMC9210450; doi:10.1126/scitranslmed.abn3041)
Supplement: Supplementary file 1 — Figs. S1 to S8 Table S1 [file scitranslmed.abn3041_sm.pdf]

Science Translational Medicine
AAAS

Supplementary Materials for

The anti-SARS-CoV-2 monoclonal antibody, bamlanivimab, minimally impacts the endogenous immune response to COVID-19 vaccination

Robert J. Benschop *et al.*

Corresponding author: Robert J. Benschop, benschop_robert@lilly.com

DOI: 10.1126/scitranslmed.abn3041

The PDF file includes:

Figs. S1 to S8
Table S1

Other Supplementary Material for this manuscript includes the following:

MDAR Reproducibility Checklist
Data file S1

Supplementary Materials for

The anti-SARS-CoV-2 monoclonal antibody, bamlanivimab, minimally impacts the endogenous immune response to COVID-19 vaccination

Robert J. Benschop[†1*], PhD, Jay L. Tuttle[†1], PhD, Lin Zhang[1], PhD, Josh Poorbaugh[1], PhD, Nicole L. Kallewaard[1], PhD, Peter Vaillancourt[1], PhD, Melissa Crisp[1], PhD, Thi Ngoc Vy Trinh[1], BSc, Joshua Joaquin Freitas[1], BSc, Stephanie Beasley[1], BSc, Montanea Daniels, BSc, Natalie Haustrup[1], PhD, Richard E. Higgs[1], MS, Ajay Nirula[1], MD, PhD Myron S. Cohen[2], MD, Mary Marovich[3] MD

*Correspondence to: benschop_robert@lilly.com

Supplementary Materials includes:

Figs. S1 to S8

Table S1

Supplementary Figures

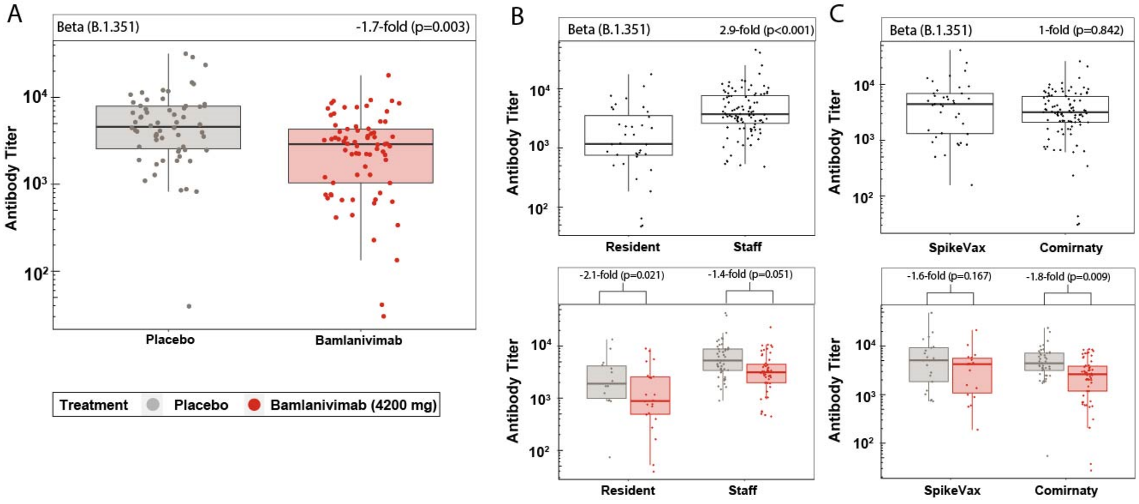

Fig. S1. Prior bamlanivimab infusion did not disparately affect endogenous antibody response to full COVID-19 vaccination in residents and staff or SpikeVax- and Comirnaty-treated patients as measured against the Beta variant. Antibody titers against the SARS-CoV-2 Beta variant (B.1.351) were tested in samples from fully vaccinated participants who (**A**) previously received either placebo (n=62) or bamlanivimab (n=73) infusion and (**B**) who were either residents (placebo n=14; bamlanivimab, n=22) or staff (placebo, n=48; bamlanivimab, n=51). (**C**) Antibody titers were also compared for recipients of the SpikeVax (placebo, n=21; bamlanivimab, n=18) or Comirnaty (placebo, n=41; bamlanivimab, n=55) mRNA vaccines. Antibody titers were rescaled after adjusting for covariates. Boxes and horizontal bars denote the interquartile range (IQR) and the median antibody titer, respectively. Length of whiskers corresponds to 1.5 times the IQR. Statistical analysis was done using a linear model (two-sided test with α level of 0.05).

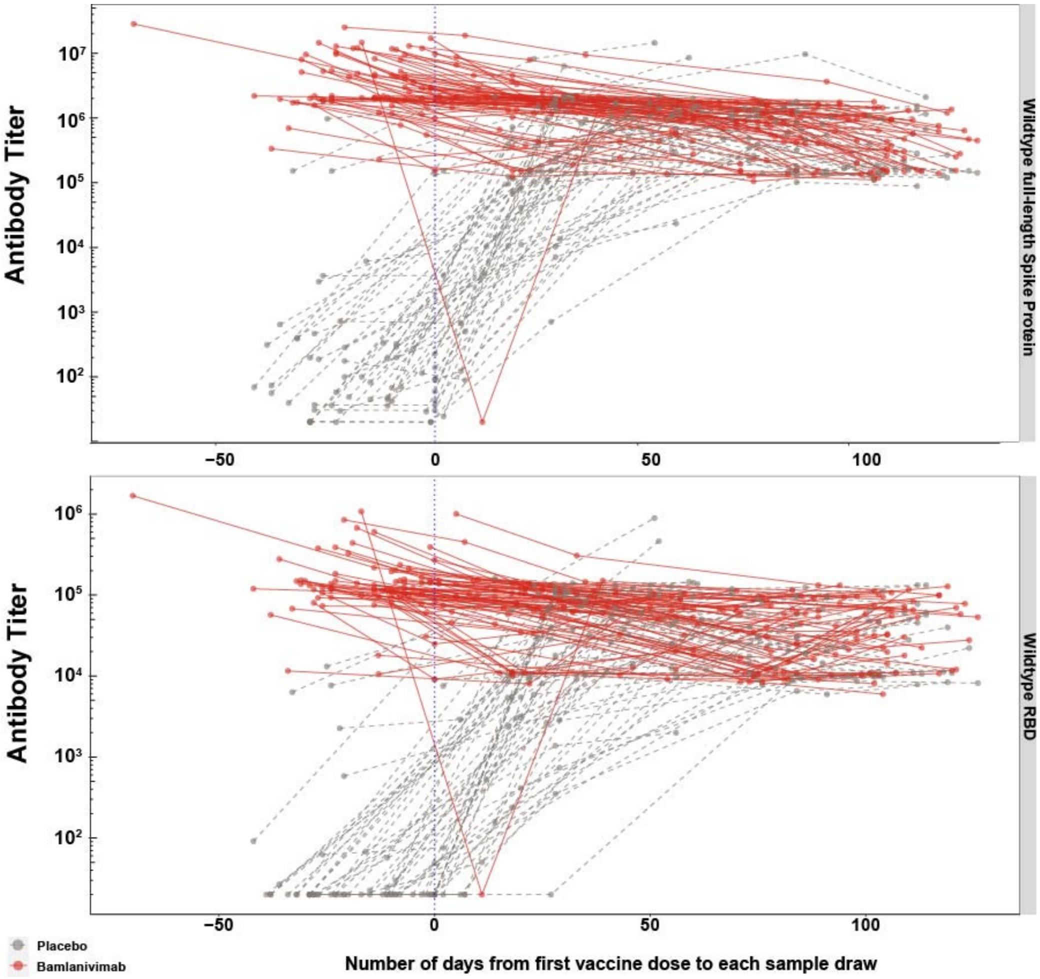

Fig. S2. Longitudinal antibody responses measured against wildtype proteins reflect both endogenous antibody response to COVID-19 vaccination and bamlanivimab. Longitudinal antibody responses were measured against wildtype full-length spike protein (top row) and wildtype receptor binding domain (RBD) (bottom row) following pre-treatment with either bamlanivimab (n=62) or placebo (n=73) and subsequent COVID-19 vaccination. Samples from the same individual are connected with dashed lines. The vertical blue dotted line denotes the timepoint where participants receive the first dose of vaccine.

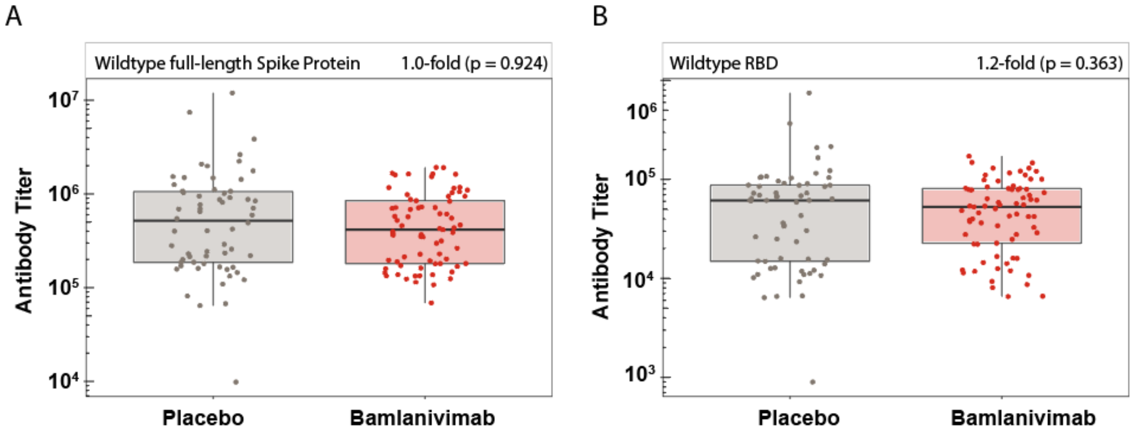

Fig. S3. Antibody response to wildtype proteins did not differ between placebo- and bamlanivimab-treated patients. Antibody titers against (**A**) wildtype full-length spike protein and (**B**) wildtype RBD were measured in samples from fully vaccinated participants who previously received either placebo (n=62) or bamlanivimab (n=73). Antibody titers were rescaled after adjusting for covariates. Boxes and horizontal bars denote the IQR and the median titer, respectively. Length of whiskers corresponds to 1.5 times the IQR. Statistical analysis was done using a linear model (two-sided test with α level of 0.05).

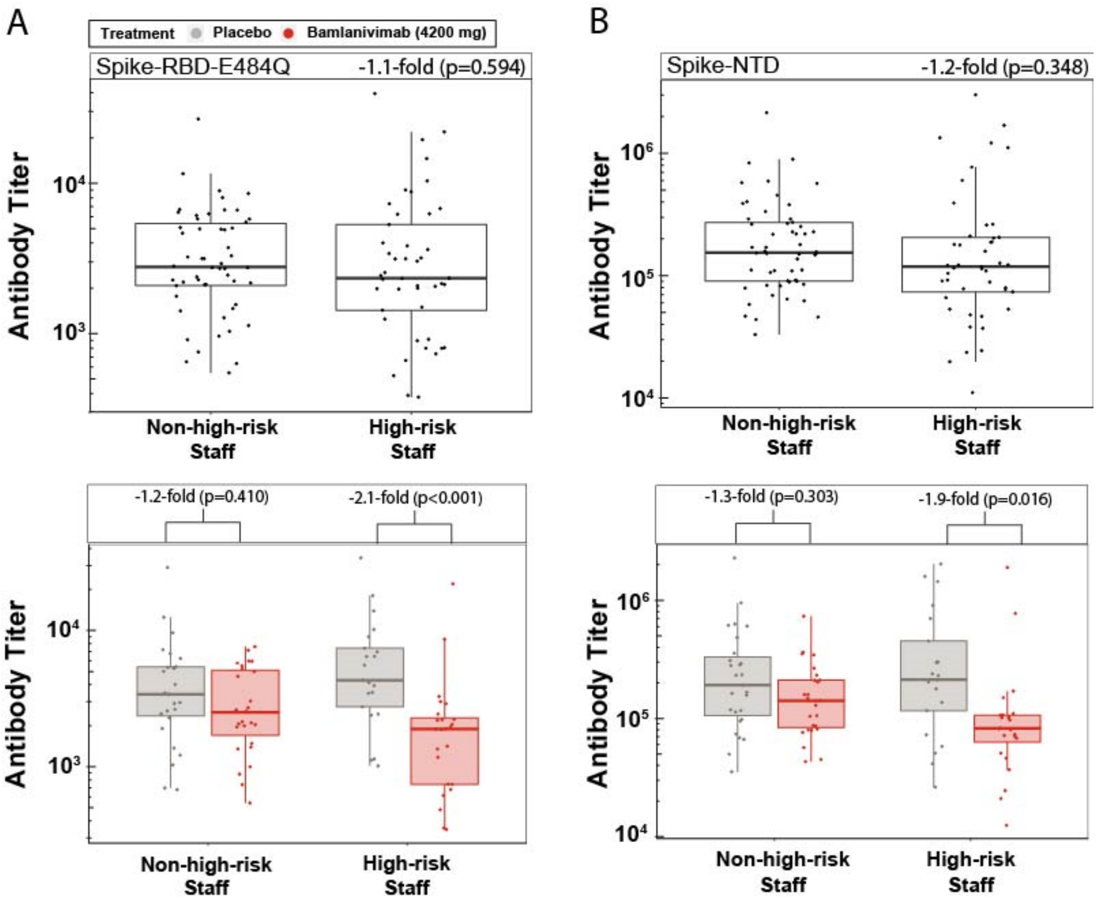

Fig. S4. Endogenous antibody response to full COVID-19 vaccination was similar for non-high-risk staff and high-risk staff against both Spike-RBD-E484Q and N-terminal domain (NTD). Antibody titers against (**A**) Spike-RBD-E484Q and (**B**) spike-NTD were compared in samples from fully vaccinated staff participants who were non-high-risk (n=54) or at high-risk (n=45) of developing severe COVID-19 (top) and further grouped by those who received placebo (non-high-risk, n=27; high-risk, n=21) or bamlanivimab (non-high-risk, n=27; high-risk, n=24) prior to vaccination (bottom). Antibody titers were rescaled after adjusting for covariates. Boxes and horizontal bars denote the IQR and the median of reciprocal half maximal inhibitory concentration (IC_{50}), respectively. Length of whiskers corresponds to 1.5 times the IQR. Statistical analysis was done using a linear model (two-sided test with α level of 0.05).

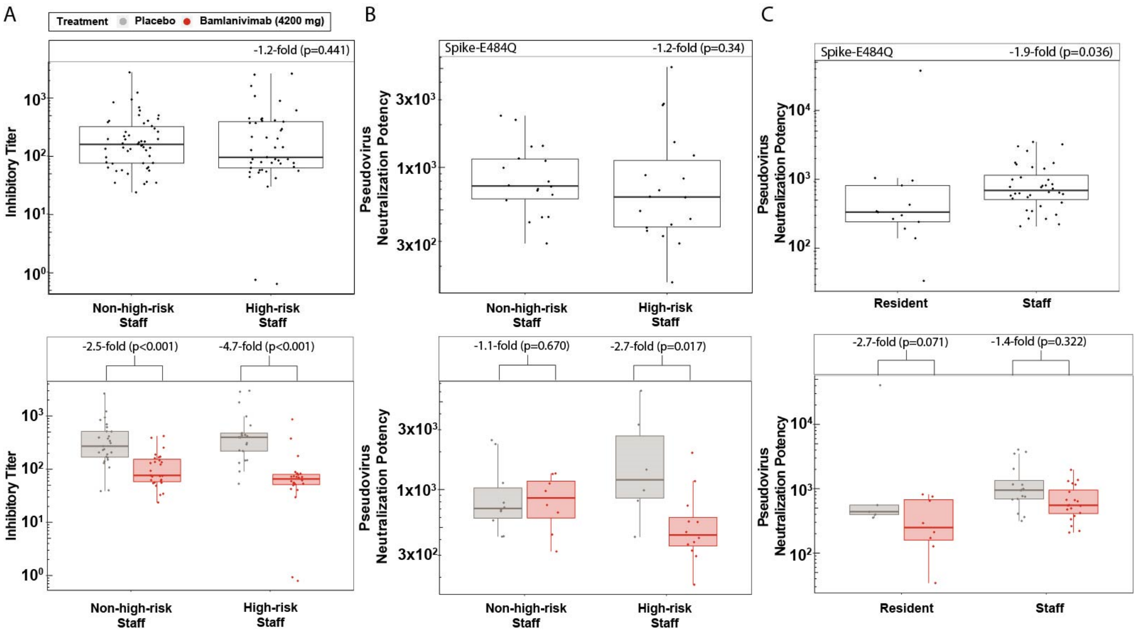

Fig. S5. The magnitude of bamlanivimab effect on inhibitory potency did not differ between non-high-risk and high-risk staff. Antibody potency ($1/IC_{50}$) was measured as (**A**) ACE2-RBD binding inhibition titer for fully vaccinated staff participants who were categorized as non-high-risk (n=54) or high-risk (n=45) (top) and further grouped by those who received placebo (non-high-risk, n=27; high-risk, n=21) or bamlanivimab (non-high-risk, n=27; high-risk, n=24) prior to vaccination. (**B**) Spike-E484Q pseudovirus neutralization was measured for samples from fully vaccinated staff participants who were categorized as non-high-risk (n=18) or high-risk (n=18) (top) and further grouped by those who received placebo (non-high-risk, n=10; high-risk, n=6) or bamlanivimab (non-high-risk, n=8; high-risk, n=12) prior to vaccination. (**C**) Spike-E484Q pseudovirus neutralization was measured for samples from participants who were resident (n=13) or staff (n=36) (top) and further grouped by those who received placebo (resident, n=5; staff, n=16) or bamlanivimab (resident, n=8; staff, n=20) (bottom). Inhibition potency was measured as $1/IC_{50}$ and adjusted for covariates and pseudovirus neutralization potency was measured as $1/NT_{50}$ and adjusted for covariates. Boxes

and horizontal bars denote the IQR and the median reciprocal of NT_{50}, respectively. Length of whiskers corresponds to 1.5 times the IQR. Statistical analysis was done using a linear model (two-sided test with α level of 0.05)

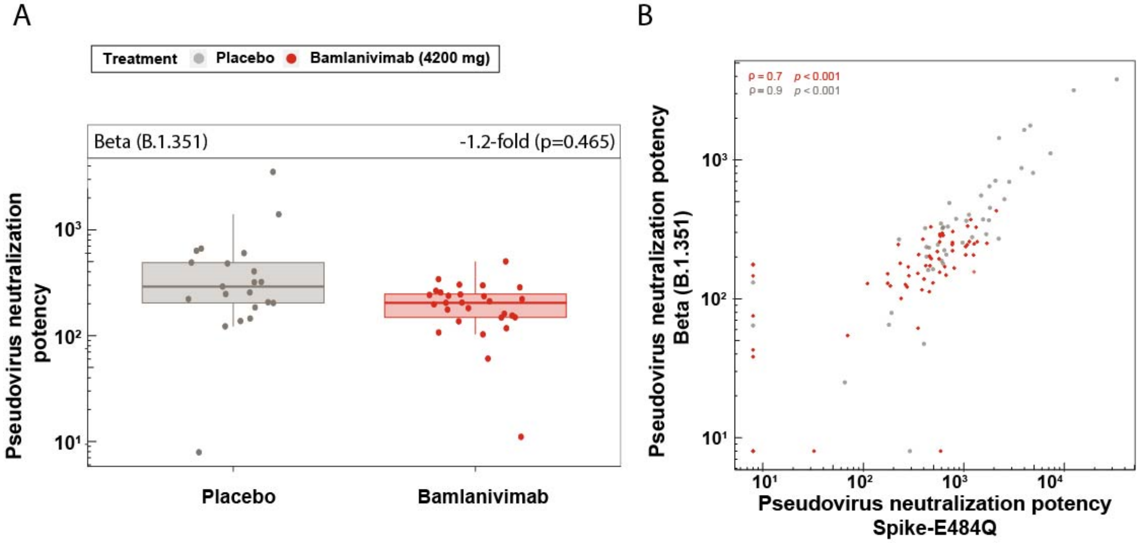

Fig. S6. Strong correlation between neutralization potency data against spike-E484Q and the Beta variant pseudoviruses. (**A**) Neutralization potency ($1/NT_{50}$) against SARS-CoV-2 beta variant (B.1.351) pseudovirus was measured for samples collected from 49 participants who received placebo (n=21) or bamlanivimab (n=28) and were subsequently fully vaccinated Boxes and horizontal bars denote the IQR and the median reciprocal NT_{50}, respectively. Length of whiskers corresponds to 1.5 times the IQR. Statistical analysis was done using a linear model (two-sided test with α level of 0.05) (**B**) The correlation plot shows neutralization potency against beta variant versus neutralization potency against spike-E484Q. Pseudovirus neutralization potency was measured as $1/NT_{50}$ and adjusted for T1 and T2 covariates. ρ represents the Spearman correlation; p represents the p-value.

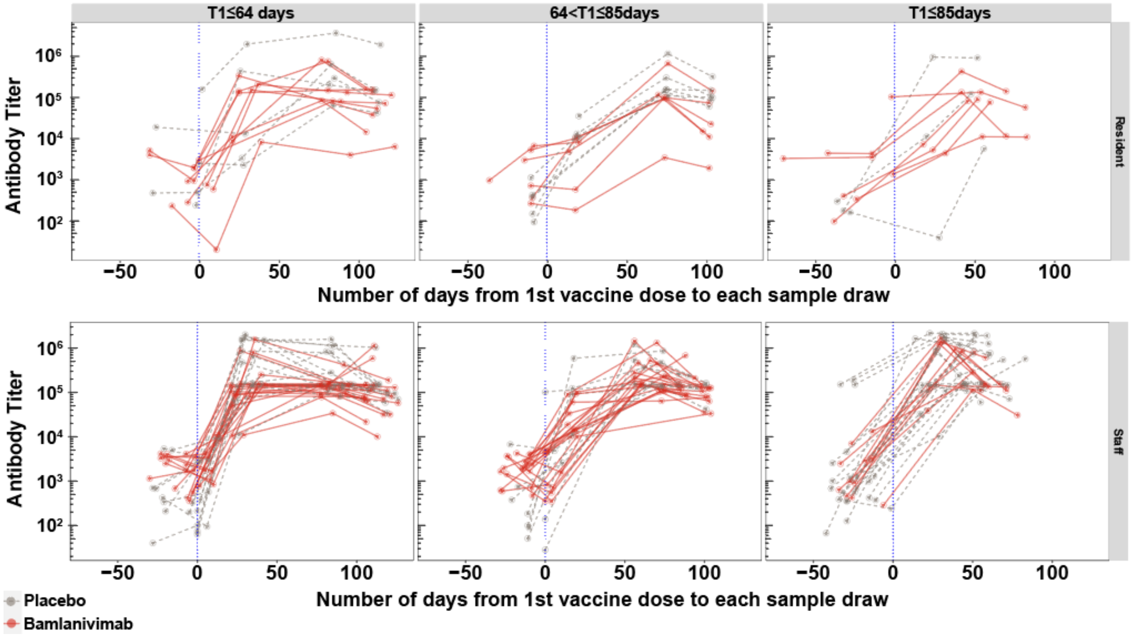

Fig. S7. Robust antibody responses against spike-NTD protein for staff and residents irrespective of prior bamlanivimab infusion. Data were arranged into three groups based on tertiles of the interval (days) between bamlanivimab or placebo infusion and first vaccine dose, T1. Three columns (left to right) correspond to T1≤64 days, 64<T1≤85 days and T1>85 days, (n=50, 42 and 43 participants) respectively. The vertical blue dotted line denotes the timepoint where participants receive the first dose of vaccine. Each dashed line connects sample titers from a single participant. The top row shows antibody titers of participants who were residents and the bottom row represents antibody titers of participants who were staff.

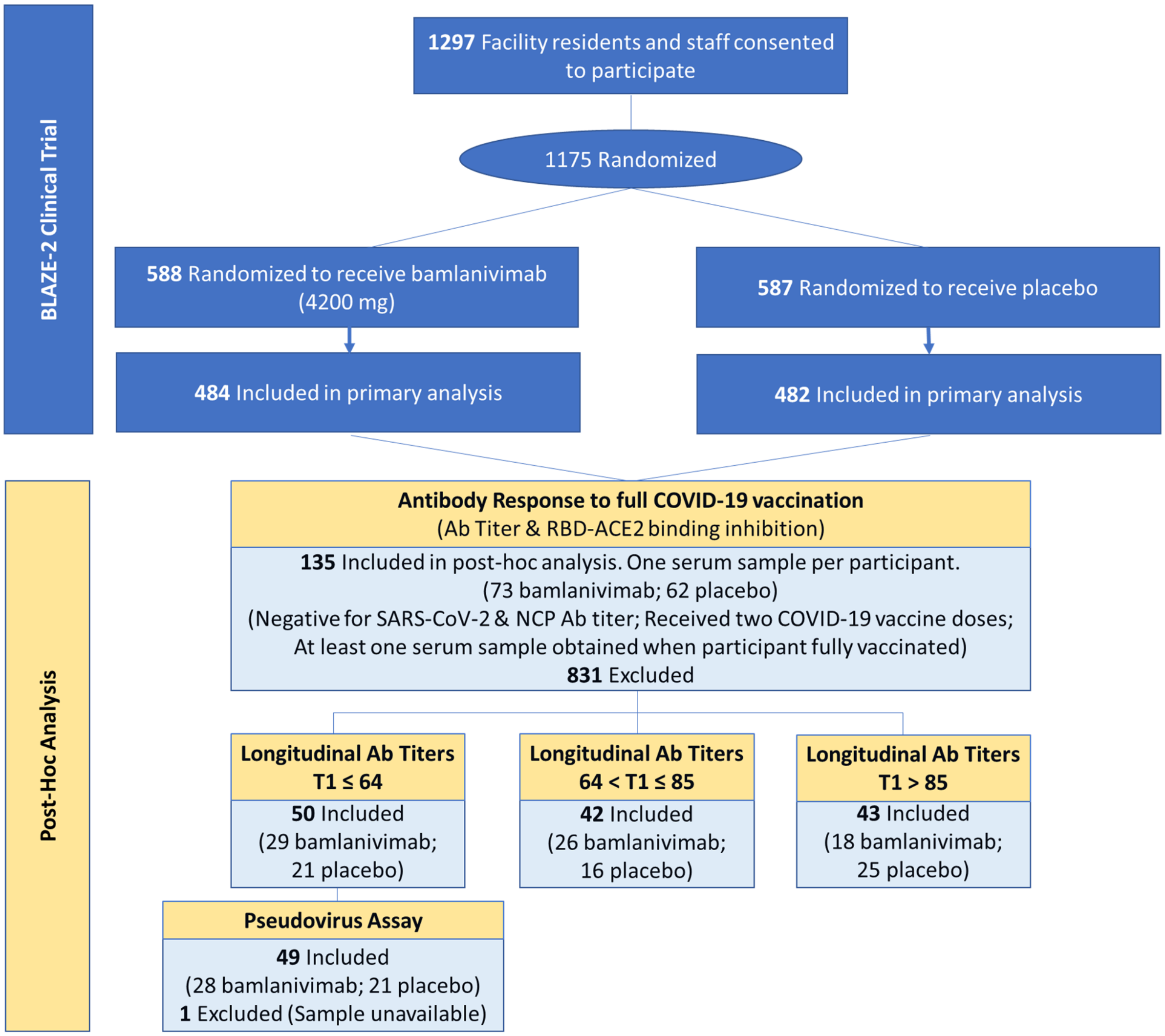

COVID-19 Vaccines: Comirnaty (Pfizer/BioNTech); SpikeVax (Moderna). T1 = interval (days) between bamlanivimab or placebo infusion and first COVID-19 vaccine dose. The CDC describes an individual as fully vaccinated after 2 weeks following the second COVID-19 vaccine dose in a 2-dose series, such as for Comirnaty or SpikeVax (23).

Fig. S8. Selection process of fully vaccinated participants for post-hoc analysis.

Table S1: Details of SARS-CoV-2 proteins used in this study.

	**Serology Assays**		
Protein	SARS-CoV-2 sequence length (amino acids)	Backbone (key mutations)	Expression
WT full-length spike	1195 (14-1208)	WT spike	CHO
WT RBD	274 (319-592)	WT RBD	CHO
Spike-RBD-E484Q	274 (319-592)	WT RBD (E484Q)	CHO
Spike-NTD	294 (14-307)	WT NTD	CHO
Beta B.1.351	274 (319-592)	WT spike (K417N/E484K/N501Y)	CHO
	**Pseudovirus Assays**		
Spike-E484Q	1256 (1-1256)	WT spike (E484Q)	NA
Beta B.1.351	1253 (1-241; 245-1256)	WT spike (L18F/D80A/D215G/del242-244/K417N/E484K/N501Y/D614G/A701V)	NA

CHO, Chinese hamster ovary; RBD, receptor binding domain; NA, Not Applicable; NCP, nucleocapsid protein; NTD, N-terminal domain; WT, wildtype (Wuhan sequence Genbank MN908947.3).

Data file S1. Individual-level data for experiments where n<20.
